# Integrated Omics Reveal Time-Resolved Insights into T4 Phage Infection of *E. coli* on Proteome and Transcriptome Levels

**DOI:** 10.3390/v14112502

**Published:** 2022-11-12

**Authors:** Maik Wolfram-Schauerte, Nadiia Pozhydaieva, Madita Viering, Timo Glatter, Katharina Höfer

**Affiliations:** Max-Planck-Institute for Terrestrial Microbiology, 35043 Marburg, Germany

**Keywords:** transcriptomics, proteomics, bacteriophage T4, *Escherichia coli*, phage infection

## Abstract

Bacteriophages are highly abundant viruses of bacteria. The major role of phages in shaping bacterial communities and their emerging medical potential as antibacterial agents has triggered a rebirth of phage research. To understand the molecular mechanisms by which phages hijack their host, omics technologies can provide novel insights into the organization of transcriptional and translational events occurring during the infection process. In this study, we apply transcriptomics and proteomics to characterize the temporal patterns of transcription and protein synthesis during the T4 phage infection of *E. coli*. We investigated the stability of *E. coli*-originated transcripts and proteins in the course of infection, identifying the degradation of *E. coli* transcripts and the preservation of the host proteome. Moreover, the correlation between the phage transcriptome and proteome reveals specific T4 phage mRNAs and proteins that are temporally decoupled, suggesting post-transcriptional and translational regulation mechanisms. This study provides the first comprehensive insights into the molecular takeover of *E. coli* by bacteriophage T4. This data set represents a valuable resource for future studies seeking to study molecular and regulatory events during infection. We created a user-friendly online tool, POTATO4, which is available to the scientific community and allows access to gene expression patterns for *E. coli* and T4 genes.

## 1. Introduction

Bacteriophages (phages) are highly abundant viruses that specifically interact with and infect bacteria. They are widespread in abundance and contribute to the largest proportion of biomass on Earth [1], thereby shaping bacterial community ecology [2]. The emergence of multi-antibiotic-resistant bacterial pathogens has led to a renaissance of phage research due to the potential application of phage-based therapies for treating bacterial infections [3,4]. Thus, the mechanisms of how phages specifically hijack their host’s gene expression machinery are of enormous current interest. A variety of model phages are subjects for studying these systems, such as bacteriophage T4 (T4 phage). The T4 phage—a member of the *Straboviridae* family—belongs to the T-even phages, infecting the prokaryotic model organism *Escherichia coli* [5]. Early studies of the T4 phage have made valuable contributions to molecular biology tools, such as T4 polynucleotide kinase, T4 DNA, and RNA ligases [6], in addition to the discovery of fundamental biological processes [5]. Among others, these include the discovery of DNA as the genetic code, messenger RNA, and understanding the role of mutations or DNA restriction and modification [7,8].

T4 phage infection is temporally fine-tuned, highly efficient, and terminates with lysis of the host from 25 to 30 min [5]. The T4 phage possesses a double-stranded DNA (dsDNA) genome of approximately 169 kb in size, encoding 288 genes [5].

Notably, the T4 phage does not possess its own gene expression machinery and thus takes over the one of its host—*E. coli*. Consequently, the T4 phage needs to reprogram the host cell to promote the expression of its genes. To promote the expression of viral genes, the T4 phage manipulates the host’s gene expression machinery using its proteins co-injected upon or expressed during infection, termed host acquisition factors (HAFs) [5,9]. These include ADP-ribosyltransferases which post-translationally modify host proteins [10,11,12,13,14], nucleases degrading host DNA and RNA [15,16], or transcription factors, which mediate the expression of phage genes from distinct promoters [5,17,18].

In combination, the action of T4-encoded HAFs leads to a temporally tightly controlled gene expression divided into an early, middle, and late infection phase [5,19]. Generally, it is accepted that *E. coli* mRNA is rapidly degraded and that host transcription is shut-off upon T4 phage infection [9,20,21]. Principally, phages are conceived to quickly takeover and dominate the transcriptome by expressing their genes in an infection-phase-specific manner and remodeling the host transcriptome to adapt to their specific needs [22,23,24,25,26]. Each infection phase is characterized by the expression of distinct sets of genes. Several studies have set out to characterize gene expression on a transcriptional level during T4 phage infection. A microarray study has focused on the time-resolved transcriptome of the T4 phage [19]. Biochemical studies have characterized specific host and phage mRNAs [9]. RNA sequencing was applied to describe the functional role of a toxin–antitoxin system during T4 infection [27]. Despite the characterization of specific gene sets, comprehensive transcriptomic analysis to investigate *E. coli* and T4 phage transcriptome is still lacking.

On the proteome level, the first attempts to elucidate the T4 phage proteome were performed via two-dimensional polyacrylamide gel electrophoresis (2D-PAGE) of phage proteins [28,29]. Applying these methods to identify small phage proteins or to characterize complex samples is time-consuming and challenging.

Recently, liquid chromatography–mass spectrometry (LC-MS)-based proteomics studies provided insights into the complex host–phage proteome (dual-proteome) rewiring of *Pseudoalteromonas* and *Bacteroidetes* and their specific phages in a time-dependent manner [30,31]. These studies shed light on essential infection regulation mechanisms from both host and virus perspectives. In addition, emerging omics technologies, such as GRAD-Seq, are powerful tools to study RNA–protein interactions during the viral predation of the bacterial cell [32]. 

In general, applying multi-omics techniques in a time-series context allows us to track the complex patterns of cellular information flow and to infer the underlying regulatory cascades [33]. This appears especially valuable in the context of phage–host interactions.

Using the power of transcriptomics and proteomics, we define the dual-phage and -host transcriptome and proteome of T4 phage infection in a time-resolved manner for the first time. Thereby, we characterized the temporal patterns of transcription and protein synthesis and their interconnection throughout T4 phage infection, not only for the T4 phage but also for the host *E. coli*. We show that most host transcripts, including tRNAs, are rapidly degraded upon infection. In contrast, four non-coding transcripts were found to be rather stable throughout infection. To the contrary, T4 phage genes are transcribed in an infection-phase-specific manner. On the proteome level, host proteins remain relatively stable, whereas the onset of phage protein synthesis occurs in distinct infection phases and corresponds to the functional protein classes needed in the respective infection phase. By comparing the time-resolved transcriptome and proteome of the T4 phage, we identified a set of genes that are transcribed early during infection but whose proteins are synthesized in the late infection phase. This indicates the presence of post-transcriptional regulatory mechanisms that control the translation of early phage mRNAs only in the late phase of infection. This work describes the first combinatorial and comprehensive study of the dual-transcriptome and -proteome of T4 phage/*E. coli* infection in a time-resolved manner. We highly appreciate all studies from the last few decades that have shaped our current understanding of T4 phage infection. Our data demonstrate that high-throughput technologies can help overcome laborious reductionist biochemical studies limited to distinct transcripts and proteins by studying a diverse population thereof at once. Moreover, by revealing the temporal coupling of RNA and protein synthesis during T4 phage infection, these data sets represent a valuable resource for future studies seeking to investigate molecular and regulatory events during infection. To enable broad community access to these data sets, we designed a web application to retrieve gene expression data for phage and/or host genes of interest called PrOteome TrAnscripTOme 4 (POTATO4).

## 2. Materials and Methods

### 2.1. Reagents

We purchased all reagents from Sigma-Aldrich (St. Louis, MO, USA) if not indicated differently.

### 2.2. Strains and Media

We obtained *Escherichia coli* strain B (*Escherichia coli* (Migula 1895)), Castellani and Chalmers 1919 (DSM 613, ATCC 11303; DSMZ, Braunschweig, Germany)) and T4 phage (Escherichia phage T4, DSM 4505; DSMZ, Braunschweig, Germany) from the DSMZ. We carried out *E. coli* strain B cultivation and T4 phage propagation in LB (Luria/Miller) medium supplemented with 1 mM CaCl_2_ and 1 mM MgCl_2_ for T4 phage infections. 

### 2.3. RNA Isolation from T4 Phage Infected E. coli

We grew a culture of *E. coli* strain B to an OD_600_ of 0.5 at 37 °C in LB (Luria/Miller) medium supplemented with 1 mM CaCl_2_ and 1 mM MgCl_2_. We added T4 phage suspension to a multiplicity of infection (MOI) of 3.1 and subsequently it grew at room temperature. We took 5 mL of culture at 0 min (before infection), 1, 4, 7, and 20 min post-infection and immediately lysed using the hot lysis method ^19^. Therefore, we incubated the culture with 1 volume of lysis solution (1% SDS, 4 mM EDTA) at 95 °C for 2 min each. We added 1 volume of water-saturated phenol (Roti-aqua phenol; Carl-Roth, Karlsruhe, Germany) to each sample and incubated at 67 °C for 10 min. We centrifuged samples at 10,000× *g* for 10 min, and then we added upper phase to 1 volume phenol/chloroform/isoamyl alcohol (Carl-Roth, Karlsruhe, Germany). We centrifuged samples again (10,000× *g*, 10 min). We precipitated the upper phase by centrifugation (14,000× *g*, 90 min, 4 °C) in the presence of 0.3 M sodium acetate and 1 volume isopropanol. We resuspended the RNA pellet in RNase-free water, and then we digested residual DNA with 2 µL DNase I (Roche, Basel, Switzerland) in 1 × DNase buffer at 37 °C for 30 min. We twice extracted the RNA with 1 volume phenol/chloroform/isoamylalcohol (Carl-Roth, Karlsruhe, Germany) and removed residual phenol by diethyl ether (ThermoFisher Scientific, Waltham, MA, USA) extraction. We again precipitated RNA in the presence of 0.3 M sodium acetate and 1 volume isopropanol by centrifugation (14,000× *g*, 4 °C, 90 min). We resuspended each RNA pellet in 50 µL RNase-free water. We performed RNA isolation in triplicates. We measured the RNA concentration using a NanoDrop 1000 Spectrophotometer (ThermoFisher Scientific, Waltham, MA, USA) and a Qubit Fluorometer (ThermoFisher Scientific, Waltham, MA, USA). We evaluated RNA integrity on a Bioanalyzer (Agilent, Santa Clara, CA, USA) using the RNA 6000 Nano Kit. 

### 2.4. Preparation of RNA-Sequencing (RNA-Seq) Libraries and Illumina Sequencing

We conducted RNA sequencing at the Deep Sequencing Core Facility of the Bioquant Heidelberg (led by D. Ibberson). We subjected 1 μg total RNA to ribosomal RNA depletion (rRNA) by Ribo-Zero rRNA Removal Kit (Gram-negative bacteria). We randomly sheared rRNA-depleted RNA in 10 μL dH_2_O at 94 °C for 10 min. We processed fragmented RNA using the NEBNext Ultra II Directional RNA Library Prep Kit for Illumina. We barcoded cDNA by PCR amplification using Illumina TruSeq adapters for Illumina. We performed further cDNA size selection in the range of 300 to 500 bp employing the Agencourt RNAClean XP kit. We examined primer-depleted cDNA by Bioanalyzer, then we measured the concentration by Qubit. We sequenced multiplexed libraries on a NextSeq 500 platform (Illumina, San Diego, CA, USA).

### 2.5. Northern Blot Analysis

We generated in vitro transcription templates for ssrA and RNAC by PCR using partially hybridizing DNA oligos (SsrA: TAATACGACTCACTATAGGACACGCCACTAACAAACTAGCCTGATTAAGTTTTAACGCTT, CGCGTGGAAGCCCTGCCTGGGGTTGAAGCGTTAAAACTTAATCAGGCTAGTTTG; RNAC: TAATACGACTCACTATAGGGTTAAAAGGCCATATCTCAACCATATCCGAACGTTCCGTCAAAAACGC, TCGATTCGAGGAAATATCTTTGCCGTAAGCCGAGTAGCGTTTTTGACGGAACGTTCGG). We transcribed radioactive RNA riboprobes for Northern blot in vitro from 1 µM DNA template in the presence of 40 mM Tris pH 8.1; 1 mM spermidine; 22 mM MgCl2; 0.01% Triton-X-100; 10 mM DTT; 5% DMSO; 0.1 mg/mL T7 RNA polymerase (purified in-house); 4 mM GTP, CTP, and GTP; 2 mM ATP; and 0.5 mCi/mL radioactive ATP (^32^P-α-ATP, 3000 Ci/mmol; Hartmann Analytics) at 37 °C for 4 h [34]. We digested and extracted in vitro transcription products using DNase I (Roche Diagnostics, Rotkreuz, Switzerland) with phenol/chloroform/isoamylalcohol (Carl Roth, Karlsruhe, Germany), as described above; then, we precipitated in the presence of 0.3 M NaOAc pH 5.5 and 1 volume isopropanol by centrifugation at 17,000× *g*, 4 °C, for 90 min. We resuspended RNA riboprobes in 50 µL MQ water. 

We analyzed 10 µg total RNA per sample via 10% denaturing polyacrylamide gel electrophoresis (5 W, 60 min). We transferred RNA to a nylon membrane (GE Healthcare) in a Trans-Blot Turbo System (Bio-Rad) in the presence of 0.5 × TBE (50 mM Tris, 50 mM boric acid, 1.25 mM EDTA) at 250 mA for 2.5 h and UV crosslinked. We pre-hybridized membrane in 20 mL ROTI^®^ Hybri-Quick (Carl Roth, Karlsruhe, Germany) for 30 min at 45 °C. We added 5 µL ^32^P-labelled RNA riboprobe to the pre-hybridized membrane and incubated at 45 °C overnight. We subsequently washed blots twice with wash solution 1 (2× SSC, 0.1% SDS) and twice with wash solution 2 (0.25 × SSC, 0.1% SDS) for 5 min each. We visualized blots with storage phosphor screens (GE Healthcare, Chicago, IL, USA) at the Amersham Typhoon imaging system (GE Healthcare, Chicago, IL, USA). We quantified band intensities using ImageLab 6.1 (Bio-Rad, Hercules, CA, USA).

### 2.6. Proteome Samples Preparation

We grew *E. coli* strain B culture in LB medium at 37 °C and 180 rpm until OD_600_ of 0.8 was reached. We performed the T4 phage infection assay at room temperature and 120 rpm. We infected cells with T4 phage at a MOI of 5. We took 2 mL samples before infection (0 min) and 1, 3, 5, 8, 12, 20, and 30 min post-infection. We immediately harvested the cells by centrifugation at 17,000× *g* for 1 min; then, we directly resuspended RT and the pellet in 200 µL hot lysis buffer (Tris-HCl pH 7.5, 1% SLS, 2 mM TCEP, 95 °C) and boiled for 10 min at 95 °C. We performed a short sonication step to degrade nucleic acids present in the samples (10 s, 20% amplitude, 0.5 pulse). We added 4 mM Iodoacetamide and incubated the samples for 30 min while protecting from light. We precipitated proteins via acetone and washed the pellets with 500 µL methanol (−80 °C) before air-drying and resuspending in 50 µL resuspension buffer (50 mM Tris-HCl, pH 7.5, 0.5% SLS). We determined the protein concentration by BCA assay (Pierce TM, BCA protein assay kit (reducing agent compatible), ThermoFisher Scientific, Waltham, MA, USA). We added 1 µg of sequencing-grade trypsin (Promega) to 20 µg of isolated proteins and digested o/n at 30 °C in the presence of 50 mM Tris-HCl, pH 7.5. We precipitated residual SLS by adding 1.5% TFA before separating precipitate by centrifugation at 4°C, 17,000× *g* for 10 min. We desalted the supernatant for mass spectrometric analysis using C_18_ solid phase columns (Chromabond C18 spin columns; Macherey Nagel, Düren, Germany).

### 2.7. Proteome LC-MS Analysis

We performed LC-MS analysis on an Exploris 480 instrument connected to an Ultimate 3000 rapid-separation liquid chromatography (RSLC) nano instrument and a nanospray flex ion source (all Thermo Scientific). We carried out peptide separation out on a reverse-phase high-performance liquid chromatography (HPLC) column (75 μm × 42 cm) packed in-house with C18 resin (2.4 μm; Dr. Maisch GmbH). For total proteome analysis, we performed peptide elution in backflush mode with a separating gradient from 98% solvent A (0.15% formic acid) and 2% solvent B (99.85% acetonitrile, 0.15% formic acid) to 25% solvent B over 40 min, followed by up to 60 min with 25% to 35% of solvent B at a flow rate of 300 nL/min. We performed label-free quantification (LFQ) data sets of total proteomes in data-dependent acquisition (DDA) mode. We acquired a high-resolution MS 1 scan at a resolution of 60,000 (at *m*/*z* 200) with a scan range from 350 to 1650 *m*/*z*, followed by MS/MS scans within 2s (Cycle 2s) of the most intense ions at a resolution of 15,000. We set charge state inclusion between 2 and 6. We set the ion accumulation time to 25 ms for MS and AUTO for MS/MS. We set the automatic gain control (AGC) to 300% for MS survey scans and 200% for MS/MS scans. The parameters of the measurements are summarized in Supplementary Table S3.

We performed DDA-LFQ analysis using MaxQuant [35] in standard settings using *E. coli* (Proteome: UP000000625) and bacteriophage T4 (Proteome: UP000009087) fusion database. We further evaluated the “proteinGroups.txt” MaxQuant output file with the SafeQuant R script updated to modify MaxQuant outputs [36]. 

### 2.8. Analysis and Visualization of RNA-Seq and Proteomics Data

We assessed the quality of reads obtained from Illumina RNA-Seq pre- and post-adapter trimming using FastQC (version 0.11.9). We processed Fastq files using the cutadapt tool (version 1.18) in order to remove reads containing Illumina TruSeq adapter sequences. We aligned reads to the genome of *E. coli* K12 (U00096.3) and bacteriophage T4 (NC_000866.4) using the hisat2 aligner (version 2.2.1) at default settings. Thereby, we successfully aligned 88.71 to 96.51% of reads to the reference genomes.

We applied Samtools (version 1.7) to select for primary alignments. We manually inspected BAM files as genomic maps using the Integrative Genomics Viewer (version 2.4.9). We quantified the reads mapped to individual features (annotated in gff3 files for U00096.3 and NC_000866.4 as gene) using featureCounts (Subread package version 2.0.1) with default settings while excluding reads overlapping multiple features.

Prior to further analysis, we manually removed *E. coli* genes annotated as rRNA (22 genes), which account for up to 0.6% of reads per sample (Supplementary Figure S1), from the counts table using R (version 4.1.2), because they were depleted from the total RNA before sequencing. We normalized the count data to transcripts per million (TPM) which allows to compare expression levels of genes between samples. We assessed sample clustering by principal component analysis (PCA) with the prcomp package. We calculated the fractions of TPM-normalized reads per sample and entity (T4 phage or *E. coli*) accordingly. We removed genes with low read counts (average read count below 1.5 across all samples) from the TPM-normalized count data, including 338 *E. coli* and 17 T4 phage genes. These genes would otherwise confuse the analysis due to low and variable read counts. We conducted further analyses in R focussed on data visualization using the pheatmap (1.0.12) and ggplot2 (3.3.6) package.

We assessed differential gene expression analysis during the early infection phase with DESeq2 by applying a Wald test at a log2fold change greater or smaller than 0 with a Benjamini–Hochberg-corrected p-value threshold of 0.05 [37]. We did not assess other infection phases by DESeq2 as dramatic changes in the host and phage transcriptome have already been recorded 4 min post-infection. We conducted assignment of differentially expressed genes to Clusters of Orthologous Groups (COGs) in R using the COG database from the National Center for Biotechnology Information (https://www.ncbi.nlm.nih.gov/research/cog/, accessed on 12 April 2021).

Different from the RNA-Seq data, we further analyzed the proteomics data based on LFQ values, as they already express the approximate abundance of the respective proteins in the sample. We also assessed sample clustering in R by PCA analysis (prcomp package) and Pearson correlation. We conducted further analyses in R focused on data visualization using the pheatmap (1.0.12) and ggplot2 (3.3.6) packages.

For both data sets, we based classification of T4 phage genes on the period of time during which the gene’s expression was below 10% its maximal detected expression (transcriptomics: TPM, proteomics: LFQ). Therefore, we initiated early gene expression is during the first 4 (transcriptomics) or 5 (proteomics) minutes of infection, middle gene expression between 4 and 7 (transcriptomics) or 5 and 8 (proteomics) minutes, and late gene expression after 7 (transcriptomics) or 8 (proteomics) minutes post-infection.

We performed comparison and integration of time-series transcriptomics and proteomics data in R using data visualization tools as described above.

## 3. Results and Discussion

### 3.1. Time-Resolved Dual-RNA-Seq of T4 Phage Infection

**Figure 1:**
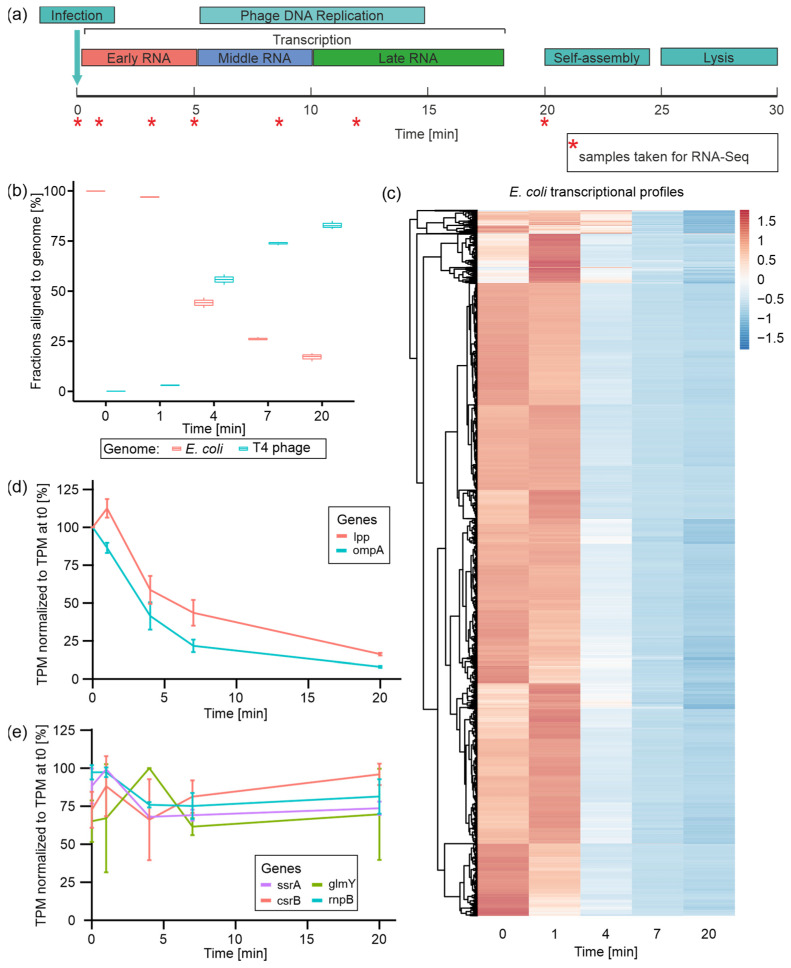
Time-resolved dual-RNA-Seq of T4 phage infection and the fate of host (*E. coli*) transcripts. (**a**) Schematic illustration of infection-phase-specific gene expression during T4 phage infection and indicated time points at which samples for dual-RNA-Seq have been taken. (**b**) Fractions of reads aligned to either *E. coli* or T4 phage genome over the time course of T4 phage infection calculated on the basis of TPM-normalized reads. (**c**) Heatmap of all *E. coli* genes’ TPM values normalized by z-score over the time course of infection. Genes are clustered according to expression profiles. (**d**) Plots of lpp and ompA mRNA levels over the time course of infection based on mean TPM values normalized to TPM value in uninfected *E. coli* (t0) [=100%] for each RNA. (**e**) Plots of transcript levels of four comparably stable *E. coli* transcripts over the time course of infection based on mean TPM values, which were normalized to TPM value in uninfected *E. coli* (t0) [=100%] for each RNA.

T4 phage infection can be divided into three temporal phases: early (0–5 min), middle (5–10 min), and late (10–20 min), which are each characterized by the expression (transcription) of distinct sets of T4 phage genes (Figure 1a) [5,19]. In order to monitor the transcriptomic changes within these phases, total RNA was isolated from uninfected *E. coli* (t0) and at 1 (t1), 4 (t4), 7 (t7), and 20 min (t20) post-T4 phage infection. For the time course of infection, we observed relatively similar yields of total RNA at all time points (Supplementary Figure S2). In order to monitor the time-resolved dual-transcriptome of phage and host during infection, rRNA-depleted total RNAs were subjected to Illumina RNA-Seq. PCA revealed the close clustering of replicates from the same time points, reflecting similar gene expression profiles except for t4, which had one outlier replicate (t4 R1) (Supplementary Figure S3).

To characterize the regulation of transcription during the course of infection, we tracked the expression of all remaining non-rRNA host genes as well as all annotated T4 phage genes (Figure 1). Following the fractions of reads per genome over the time course of infection, it becomes evident that the T4 phage quickly starts to dominate the transcriptome during the first seven minutes of infection (73.8% of T4 phage reads at t7) (Figure 1b). At the end of infection, the overall fraction of phage reads amounts to 82.8% (Figure 1b), similar to what was reported by Laub and colleagues [27]. In parallel, *E. coli* transcripts rapidly decline in abundance, which has been reported in several transcriptome studies [9,16,38], amounting to less than 20% among all reads at t20 (Figure 1b). This fast and nearly complete takeover of the non-rRNA transcriptome by T4 phage is a phenomenon commonly observed in other phage–host interactions, and is conceived to liberate nucleotide building blocks for phage transcription, DNA replication, or ATP metabolism [22,23,26,39].

### 3.2. E. coli Transcript Degradation Is Initiated during the First 4 min of T4 Phage Infection 

During T4 phage infection, *E. coli* gene expression is globally shut off, whilst existing host transcripts are predominantly degraded [40]. This conception is mainly based on valuable studies describing the degradation of small subsets of host transcripts using elaborate rifampicin and Northern blot assays [9]. Thus, our dual-transcriptome approach provides the first comprehensive insights into all *E. coli* transcripts during T4 phage infection based on a single experiment.

Initially, we tracked the levels of all *E. coli* transcripts over the time course of infection (Figure 1c). For the vast majority of *E. coli* genes, transcript levels remain stable during 1 min post-infection—regardless of whether these transcripts are highly abundant or not. Subsequently, the levels of most transcripts drop immensely at 4 min post-infection and reach their minimum at the end of infection (t20). Only for a smaller set of genes we observed a slower decrease in transcript levels where the drop at t4 is less intense. This overall decline in host transcript levels is in good agreement with the decline of host mRNA read fractions observed throughout Pseudomonas phage LUZ19 and Acinetobacter baumannii phage *phiAbp1* infections [22,23]. During T4 phage infection, we observed an immense degree of host transcript degradation (75% reduction in host reads) already 7 min post-infection, representing the efficiency of phage-induced host transcriptional takeover (Figure 1b,c, Supplementary Figure S4). Exemplarily, the mRNAs transcribed from the *lpp* and *ompA* genes are quite stable mRNAs in *E. coli*, with half-lives of 31 and 30 min, respectively [9]. Using rifampicin assays, Ueno and Yonesaki reported their rapid destabilization during T4 phage infection, reducing their half-lives to 2.3 and 2.5 min, respectively [9]. In accordance with these studies, our RNA-Seq data reveal a decline of transcript levels by approximately 50% for both lpp and ompA mRNA within the first 4 min of infection (Figure 1d). Interestingly, host-RNA degradation concerns not only mRNAs but also non-coding RNAs and transfer RNAs (tRNAs). For *E. coli* tRNAs, we detected steady and even increasing transcript levels within the first minute of infection, followed by a steep decrease in their abundances towards the end of infection (Supplementary Figure S5). This indicates predominant host tRNA decay during T4 phage infection, which has been described for a T4-like vibriophage so far [41]. Yang et al. speculate that early phage genes are translated using the host tRNA pool, which is successively degraded during infection followed by the transcription of phage tRNAs. These contribute to late phage mRNA translation, which prefers the phage tRNA code. Similar to the T4-like vibriophage, T4 might use host tRNAs for the initial translation of early mRNAs and subsequently as a nucleotide resource.

Despite observing massive host-RNA degradation, we questioned whether some *E. coli* transcripts might be comparably stable throughout infection. Therefore, we selected host genes with a mean expression accounting for at least 10 TPM and with at least 80% TPM at t20 compared with t0. Using these criteria, we identified four comparably stable transcripts (Figure 1e). Equal read distributions at these genes over all time points indicate that the determined TPM levels are not derived from degradation fragments accumulated during infection but rather from similar transcripts as detected before infection (Supplementary Figure S6). Among those stable host transcripts, we identified the highly abundant (mean of 67,858 TPM) transfer messenger RNA (tmRNA) SsrA, which was validated by Northern blotting (Supplementary Figure S7a). SsrA plays an important role in ribosome rescue and protein degradation [42,43]. Moreover, functional SsrA is required to induce the prophage of bacteriophage Mu and may act as a sensor for prophage activation [44]. This tmRNA has been reported as a highly stable RNA with a half-life of 89 min [45], which may explain its observed stability even during T4 phage infection. However, *E. coli* tRNAs that have similar RNA stability characteristics to SsrA are specifically degraded during T4 phage infection (Supplementary Figure S5a). One may speculate that SsrA is constitutively required during infection to keep the maximal amount of ribosomes available for the translation of T4 phage mRNAs, finally enabling the fast and efficient infection process. 

Furthermore, the non-coding Rnase P RNA encoded by the *rnpB* gene was found to be relatively stable during infection. This RNA serves as a catalytic center in Rnase P [46], which plays a role in the maturation of tRNA by trimming the 5′-ends of tRNA precursors [47]. Potentially, this catalytic RNA may play a role in phage tRNA processing. Furthermore, GlmY (from *glmY* gene) and CsrB (from *csrB* gene) were identified as stable host transcripts, which are small regulatory RNAs (sRNAs) that affect gene expression by stabilizing or destabilizing target mRNAs through RNA–RNA and RNA–protein interactions, respectively (Figure 1e, Supplementary Figure S6b,c) [48,49]. Surprisingly, both sRNAs are usually unstable in exponentially growing *E. coli*, with half-lives of around 1.5 min [48,49]. As CsrB sequesters CsrA, a global activator of glycolysis (Sabnis et al., 1995), it stands to reason that the host may downregulate glycolysis to inhibit phage replication by temporally stabilizing CsrB. Additionally, the role of GlmY stabilization remains elusive. Its function is linked to a susceptibility to cell envelope stress [50], which could be a strategy employed by the host to strengthen the cell envelope and protect against infection. However, due to predominant host mRNA degradation, the potential actions of these sRNAs may be aborted as a consequence of the lack of target RNAs.

In summary, apart from a few, surprisingly yet unreported stable RNAs during T4 phage infection, we observed global host transcript degradation.

As we also observed that a fraction of *E. coli* genes exhibit higher TPM values at t1 compared with t0 (Figure 1c), we speculated that these genes might be—temporarily—significantly upregulated as a response to the invading phage during the early phase of infection. Using DESeq2, we detected 1050 genes differentially expressed at t1 compared with t0 (log2 fold change > 0 or <0; adjusted *p*-value ≤ 0.05) (Supplementary Figure S8a, Supplementary Table S1). A total of 505 of these genes were upregulated, predominantly fulfilling functions in transcription, energy production, and conversion as well as carbohydrate transport and metabolism (Supplementary Figure S8b). We also observed the upregulation of the two host genes constituting the mcrBC system, which is known to counteract cytosine hydroxymethylation of T4 phage DNA as a defense mechanism [51]. Other phage defense systems present in this host’s genome, including the *mazF*, *9aze* [52] and *lit* [53] genes, which were not differentially expressed. The set of 545 downregulated host genes is dominated by functions in translation and ribosome biogenesis, amino acid transport, and metabolism, as well as energy production and conversion (Supplementary Figure S8b). This initial gene regulatory alteration could resemble a host response to infection, a phage-induced change, or a combination thereof. Considering gene expression in other phages, one may speculate that these changes may originate from initial host cell reprogramming by the phage in order to create an optimal environment for phage infection [22,23,24,26].

In conclusion, we suggest that the host may initially try to adapt to T4 phage infection by gene expression changes, which are quickly interfered with by the phage that rapidly initiates the degradation of the vast majority of host transcripts.

### 3.3. T4 Phage Transcription Is Actively Controlled in an Infection-Phase-Dependent Manner

**Figure 2:**
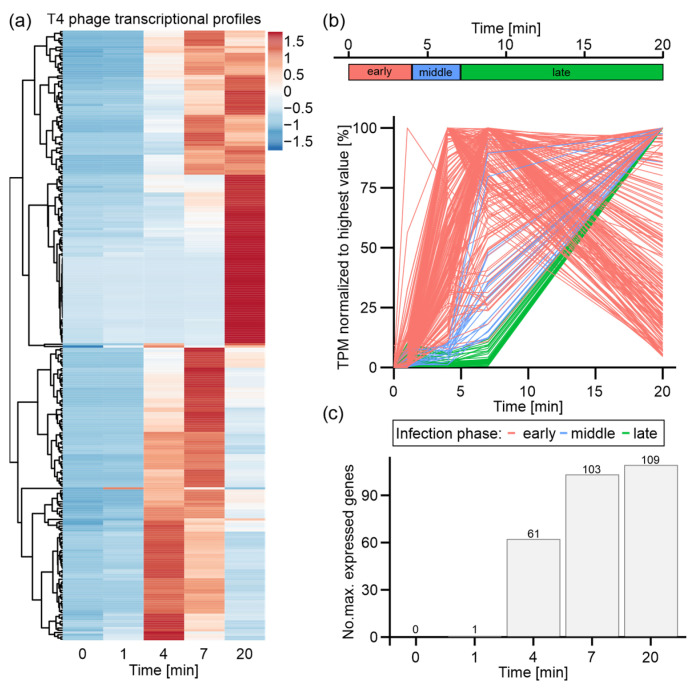
Expression and classification of T4 phage genes during infection of *E. coli*. T4 phage genes with mean TPM value below 1 were excluded from all analyses. (**a**) Heatmap of all T4 phage genes over time course of infection based on z-score-normalized TPM values. (**b**) Criteria for the classification of T4 phage genes: early (red), middle (blue), and late (green) based on the time frame of the onset of expression and maximal expression values (upper panel). Plots of all T4 phage genes over time course of infection based on mean TPM values normalized to the highest mean TPM value of each gene (lower panel). Genes colored by classification based on criteria depicted in upper panel. (**c**) Quantification of maximum expression of T4 phage genes per time point of infection.

Apart from host transcriptional alterations, we obtained a time-resolved picture of T4 phage gene expression (Figure 2). We observed distinct gene expression patterns for different sets of T4 phage genes (Figure 2a). A large group of T4 phage genes (109 genes) is most strongly expressed at the end of infection, whereas the maximal transcript levels of similarly sized sets of genes are detected at t4 (62 genes) or t7 (103 genes) in Figure 2c. Furthermore, the expression onsets, the initiation of degradation, and the decline of transcript levels vary highly among phage transcripts. Altogether, these findings vividly demonstrate the different classes of T4 phage genes, which have previously been reported [5,19]. Based on an already existing criterion for the classification of T4 phage genes [19], we defined criteria suiting our choice of time points for dual-RNA-Seq. Therefore, we classified a T4 phage gene based on its onset of expression, which resembles the period of time during which TPM values for a distinct gene are below 10% of its maximal TPM value. Accordingly, the expression of early genes starts during the first four minutes of infection (t0–t4), followed by middle genes (t4–t7) and late genes (t7–t20) (Figure 2b).

Based on this classification, most early genes show highest expression levels at t4 or t7, which decline towards t20 (Figure 2b). In total, 215 early genes were classified, which are predominantly associated with host cell reprogramming, DNA and RNA degradation, and some metabolic processes, as represented by gene functions and gene ontology (Supplementary Table S2). Additionally, the genes for the eight T4 phage-encoded tRNAs were classified as early genes. Intriguingly, this is the first characterization of all eight T4 tRNAs, as a previous microarray study had only focused on tRNA 2, 3, and 4 [19]. It appears that the expression of these eight tRNAs is initiated early during infection and continuously increases during the course of infection (Supplementary Figure S5b).

Middle and late genes are maximally expressed at t20 and differ by the onset of expression (either just after t4 or t7, respectively). We classified 21 middle and 38 late T4 phage genes, which overall encode structural phage proteins, viral release factors, and proteins associated with DNA replication. Overall, middle and late genes mediate processes that are important at the end of infection prior to phage particle release from the host cell [5,54] (Supplementary Table S2). Moreover, we also classified the small regulatory RNAs RNAC and RNAD – RNAs of yet unknown functions – as late RNAs, as reported earlier [19], and validated them by our Northern blot assay (Supplementary Figure S7b).

In summary, we detected the well-established infection-phase-specific T4 phage gene expression that progresses from host cell reprogramming over DNA replication to phage assembly and host cell lysis, which is a common feature shared by other transcriptionally characterized phages [19,22,23].

### 3.4. Time-Resolved Dual-Proteome of T4 Phage Infection

**Figure 3:**
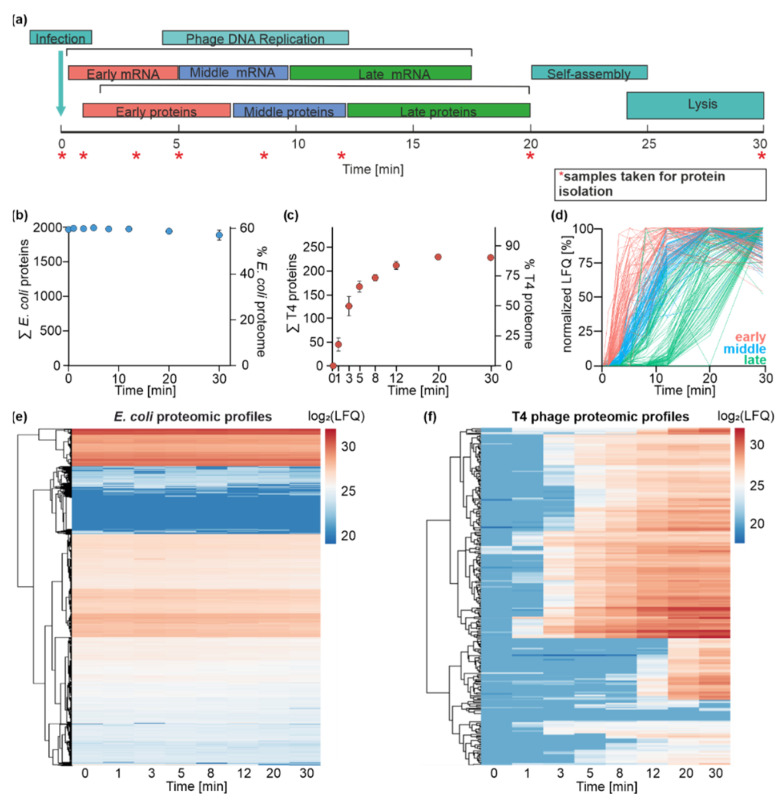
Time-resolved dual-proteome of T4 phage infection of *E. coli*. (**a**) Schematic illustration of infection-phase-specific gene expression during T4 phage infection. The time points at which samples were collected for the dual-proteome are indicated with an asterisk. (**b**) Monitoring of *E. coli* proteins over the time course of infection. The left *y*-axis identifies the absolute number of detected proteins, and the right *y*-axis depicts the coverage of the annotated *E. coli* proteome (*n* = 3). *E. coli* proteome is not significantly altered throughout the infection process. (**c**) Monitoring of T4 phage proteins over the time course of infection. The left *y*-axis identifies the absolute number of detected proteins, and the right *y*-axis depicts the coverage of the annotated T4 phage proteome (*n* = 3). The diversity of the T4 phage proteome increases throughout the infection. After 20 min, 85% of annotated T4 phage proteins are identified. (**d**) Assignment of T4 phage proteins to specific temporal groups. Criteria for the classification of T4 phage proteins are shown in the graph: early (red), middle (blue), and late (green) based on the time frame of the onset of protein detection and its maximal abundance values. Plots of all T4 phage proteins over the time course of infection based on mean MaxQuant LFQ values normalized to the highest mean LFQ value of each protein. Proteins are colored by classification based on their temporal appearance (*n* = 3). (**e**) Heatmap representation of the clustered *E. coli* protein abundance over the time course of infection based on log2-transformed LFQ values (*n* = 3). (**f**) Heatmap representation of T4 phage protein log2-LFQ intensity over the time course of infection (*n* = 3).

The dynamics of the transcriptome usually correlate with the changes in the composition of the proteome. To systematically track the time course of changes in the quantity of proteins during the T4 phage infection of *E. coli*, we set out to apply proteomics. For this purpose, the total proteome was isolated from uninfected *E. coli* (t0) and 1 (t1), 3 (t3), 5 (t5), 8 (t8), 12 (t12), 20 (t20), and 30 (t30) min post-infection with T4 phage in biological triplicates (R1-R3) (Figure 3a). These time points cover the same infection phases as the ones analyzed in the transcriptomics experiment (t0–t20) and additionally include time point t30. The rationale of the latter is to capture the maximal possible number of T4 phage proteins via proteomics. 

Our proteomics workflow yielded a label-free quantified data set, allowing for the identification of 2572 proteins in total over the entire time course of infection. A total of 2326 proteins were assigned to *E. coli* and 246 to T4 phage. This results in 60% coverage of the known *E. coli* proteins and 85% of the annotated T4 phage proteins (Figure 3b,c) [5,55]. PCA and Pearson correlation revealed close clustering within biological replicates of the same time point, indicating the high consistency of the proteomics data (Supplementary Figures S9a, S10 and S11).

In order to determine the abundance of viral proteins during infection, we calculated the fractions of the LFQs contributed by T4 phage and *E. coli* over the time course of infection (Supplementary Figure S9b). During the infection, the LFQs are predominantly contributed by *E. coli* proteins. The fraction of the signals contributed by viral proteins increases throughout the course of infection, reaching its maximum of 14% at 30 min post-infection.

### 3.5. E. coli Proteome Remains Stable during T4 Phage Infection

Transcriptome analysis has shown that *E. coli* transcripts are predominantly degraded in response to T4 phage infection. To analyze the host’s response to phage infection on the level of the proteome, we examined the abundance of individual *E. coli* proteins. 

In contrast to *E. coli* transcripts, we did not observe significant changes in *E. coli* protein abundance, indicating their stability throughout infection (Figure 3e), which was additionally confirmed by an SDS-PAGE analysis of the total proteome throughout infection (Supplementary Figure S12). 

The first attempts to analyze the stability of host proteins during the infection with T4 phage were performed by Simon and Tomczak in 1978 [56]. They reported that the maintenance of protein stability is a specific feature of T4 phage infection. Other *E. coli* phages, such as T7 and T5, utilize their own RNA polymerases for viral gene expression; however, T4 phage uses the *E. coli* RNA polymerase for this purpose. This dependence of T4 phage on host proteins might result in the preservation of the host proteome during the infection, which can be observed in our data set [57,58,59]. The hijacking of crucial *E. coli* protein complexes, such as ribosomes or the RNA polymerase, allows T4 to start with the rapid production of its transcripts and proteins and to lyse its host in up to 30 min.

### 3.6. T4 Phage Protein Synthesis Is Temporally and Functionally Regulated

In contrast to the *E. coli* proteome, we detected a highly dynamic T4 phage proteome throughout infection (Figure 3c,f). Viral proteins were observed already 1 min post-infection, and the number of detected T4 phage proteins increased exponentially until t12. The maximal number of identified T4 phage proteins was reached already at 20 min post-infection and remained stable until t30, confirming the detection of the maximal number of phage proteins. We obtained a coverage of up to 85% of the T4 phage genome. Proteins that were not identified in our proteomics study were classified as early transcripts in our transcriptome data set (Supplementary Table S2) and belong to a class of uncharacterized/hypothetical proteins (e.g., ProtID P13322, ProtID P13322). The lack of detection for some proteins might be due to their low expression levels during the early phase of infection or their small sizes (e.g., ProtID P39249 (5 kDa)), which limit their detection via LC-MS. 

Our dual-proteome data set revealed the time-resolved onset of viral protein synthesis, confirming that T4 phage protein synthesis is a temporally highly regulated process (Figure 3e). A similar temporal regulation of protein biosynthesis has been observed in other studies characterizing viral proteomes, e.g., for marine phages [30,31]. 

In order to distinguish between temporal T4 phage protein classes (early (t0–t5), middle (t5–t8), and late (t8–t30)), we applied the same criteria as used for the classification of T4 phage transcripts. Briefly, we assigned T4 phage proteins to temporal groups based on the onset of protein detection (>10% of the maximal LFQ value of a specific protein). Based on this classification, we identified 61 early, 79 middle, and 105 late proteins (Figure 3d). We speculated that the temporal appearance of proteins could be linked to their functions, which we already observed on a transcriptome level. 

Among 61 assigned early T4 phage proteins, T4 phage-encoded HAFs and regulatory proteins were identified, including ModA, ModB, MotB, and Dmd [10,18,60,61]. This observation is consistent with the concept that early infection phases are dominated by host cell reprogramming/adaptation processes [19,22,26] (Supplementary Table S2). 

Furthermore, host-defense mechanisms are activated during the early phase of T4 phage infection. These include the inactivation of the host-derived proteases to prevent the degradation of the viral proteome [62]. In the genome of *E. coli,* more than 60 proteases and peptidases are encoded. In our data set, we identified 32 proteases and peptidases (Supplementary Table S3) expressed throughout phage infection. Most of them are involved in protein maturation and the cleavage of protein signal sequences and are described to be not of particular interest for phage infection [63,64]. However, two detected proteases were reported as crucial for infection: Lon protease and its predicted and uncharacterized homolog, LonH. In *E. coli*, Lon is responsible for cellular homeostasis, protein quality control, and metabolic regulation; however, it is also responsible for the selective degradation of short-lived regulatory proteins and abnormal proteins, such as the proteins of bacteriophages [65,66]. Our proteomics data reveal that Lon and LonH are consistently abundant during infection (Supplementary Table S3).

Nevertheless, T4 phage escapes Lon and potentially LonH activity by the expression of the Pin protein. The T4 phage Pin protein is described as a specific inhibitor of Lon [67]. The interaction of Pin and Lon leads to the complete inhibition of the degradation of T4 phage proteins [56]. Our data show that protease inhibitor Pin can be detected 3 min post-infection and that Pin appears to be 6.5-fold more abundant than Lon based on average LFQ values throughout infection (Supplementary Table S3). This provides novel insights into the regulation of host proteases by the T4 phage. Possibly by producing high amounts of antihost factors, such as Pin, the host’s phage defense by proteases is prevented.

A few T4 phage proteins involved in the metabolism of nucleic acids are also synthesized in the early phase of infection, such as the nucleases RegB and MobB, followed by other nucleases appearing at subsequent time points of infection (Supplementary Table S3). Nucleases are involved in the degradation of the host transcripts, which can be confirmed by our transcriptomics data set (Figure 1c). Their activity leads to the generation of building blocks for the synthesis of viral DNA and RNA, restricting *E. coli* gene expression at the same time, as described above. 

Besides HAFs and regulatory proteins, most early proteins (~70%) belong to a class of uncharacterized/hypothetical proteins. These uncharacterized proteins are primarily encoded in intergenic regions of the T4 phage genome [68]. Nevertheless, based on the functions of other early proteins, one may speculate that some of these uncharacterized proteins might be involved in host-hijacking or host-defense processes. 

In total, 79 T4 phage proteins meet the criteria for middle proteins (Supplementary Table S3). At the middle phase of the infection, the number of proteins that are involved in T4 phage DNA replication (gene 61 (DNA primase), gene 43 (DNA polymerase)) and its protection against host nucleases (a-gt, b-gt) strongly increases. This indicates that infection proceeds from host adaptation (early phase) towards phage DNA replication (middle phase).

Finally, 105 T4 proteins were classified as late proteins. This class is mainly comprised highly abundant structural proteins and proteins involved in phage assembly and packaging, whose activity results in the formation and release of T4 phage progeny upon host lysis (Supplementary Figure S13, Supplementary Table S3). 

Altogether, the data collected in this study confirm the previously assumed temporally resolved and overall highly organized T4 phage protein biosynthesis [29]. The temporal organization of the T4 phage proteome matches a clear pattern from early host cell reprogramming to DNA replication in the middle infection phase, ending with phage assembly and release. In addition, a direct link between the point in time of protein appearance and its function can be made. This fact might be beneficial for the elucidation of the biological functions of numerous uncharacterized T4 phage proteins that appear at various stages of infection.

### 3.7. Correlation of Transcriptomics and Proteomics Data Implicates Post-Transcriptional Mechanisms Governing T4 Phage Gene Expression

Our dual-transcriptomics and -proteomics study of T4 phage infection with *E. coli* revealed the temporal control of RNA transcription and degradation as well as protein synthesis during infection. On the transcriptomic level, it stands out that host RNAs are predominantly degraded during infection, including the large group of host mRNAs, whereas host proteins remained rather stable. Thus, host mRNA translation appears to be rapidly shut-off upon T4 phage infection. Mechanistically, it seems reasonable that the T4 phage takes over the entire transcription capacities of the host whilst harnessing the existing cellular protein machinery, such as the gene expression apparatus for its propagation. 

**Figure 4:**
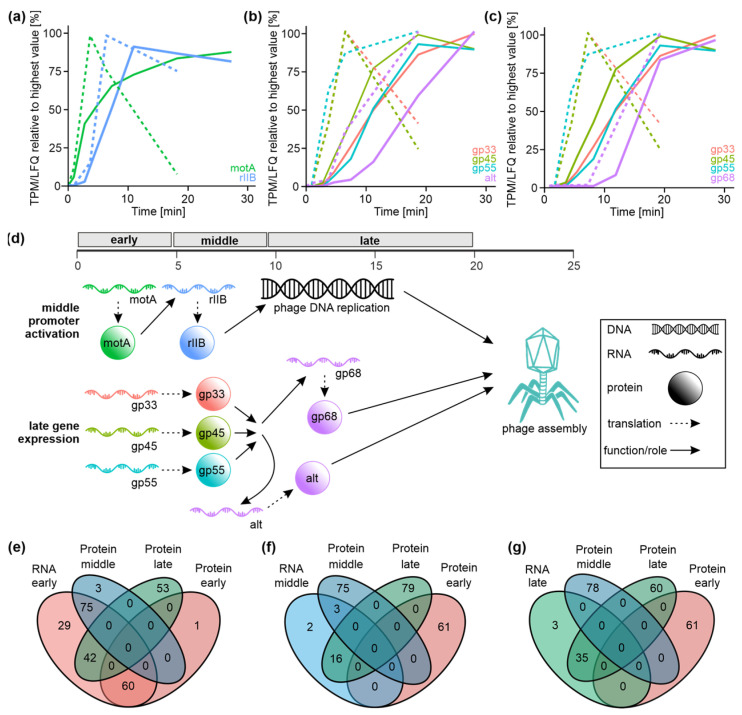
Analysis of correlation of transcriptome and proteome data of T4 phage infection. (**a**–**c**) Examples of the gene expression regulation during T4 phage infection based on the transcriptome (dashed line) and proteome data (continuous line). (**a**) Example of MotA expression which activates middle transcription of the *rIIB* gene. (**b**,**c**) Expression of the activators of late transcription—gp33, gp45 and gp55 triggers late expression of Alt (**b**) and gp68 (**c**). (**d**) Schematic representation of selected gene regulatory cascades during T4 phage infection. A similar color code as shown in (**a**–**c**) was applied. Infection-phase-specific transcripts and proteins are shown in the order of their appearance during infection based on the data shown in (**a**–**c**). Arrows indicate the transition between gene regulatory and molecular events. (**e**–**g**) Venn diagrams displaying overlaps of T4 phage gene classes based on proteome data with either early genes (**e**), middle genes (**f**) or late genes (**g**) derived from transcriptome data set.

Whilst *E. coli* gene expression lacks temporal up- and downregulation patterns during infection, we observed the synthesis and degradation of T4 phage transcripts as well as the controlled onset of T4 phage protein synthesis in distinct infection phases. The power of our dual-omics approach not only enabled us to track time-resolved gene expression during infection but also allowed us to infer specific already-studied gene regulatory cascades within T4 phage infection. Here, we demonstrate the temporal order of T4 phage gene expression events for specific examples well-described in the literature. Conceptually, early T4 phage gene products mediate the transition to middle gene expression, which in turn activates the late infection phase (Figure 4).

For instance, the induction of the expression from T4 middle promoters is partially mediated by the MotA protein—an early gene product also classified as such in our data [69,70]. We observed the coordinated onset of *motA* gene transcription (transcriptome, t1) and translation (proteome, t1) early during infection followed by the expression onset of the *rIIB* gene—controlled by a T4 middle promoter—approximately 3 min afterwards (transcriptome, t4; proteome, t5) (Figure 4a). Similarly, production of AsiA—the co-activator of middle transcription during T4 phage infection [17,71]—is detected at early time points in our data sets (transcriptome, t1; proteome, t1-t3). On the other hand, late T4 phage transcription requires a sigma factor composed of the proteins gp55 and gp33 [72,73]. In addition to the gp55–gp33 complex, the transcriptional activator gp45 is required to initiate the transcription from the late phage promoters [74,75]. As a consequence, late gene products are formed, which mainly fulfil roles in DNA packaging and capsid assembly. On both transcriptome and proteome levels, we detected the expression of the activators of late transcription—gp33, gp45, and gp55 (each: transcriptome, t1; proteome, t5)—in the early and middle phases of infection (Figure 4b,c). Upon the presence of all three gene products, we observed the expression of late genes, such as *alt* (transcriptome, t7; proteome, t8), whose gene product is part of the mature T4 phage progeny [76] (Figure 4b) or *68* (transcriptome, t7; proteome, t12), encoding a structural phage head protein [77] (Figure 4c). This emphasizes that our time-resolved dual-transcriptomics and -proteomics approach can reproduce T4 phage gene regulatory cascades (Figure 4d), which in combination contribute to phage assembly and release. Importantly, the above-described gene expression cascades were reported in previous studies with the focus on these particular proteins only [69,70,74,75]. In contrast to these highly valuable but laborious biochemical studies, our data enable the investigation of possibly any gene expression cascade during T4 phage infection simultaneously on the transcriptome and proteome levels. To enable broad community access to these correlated data sets, we designed a user-friendly web application (POTATO4) to retrieve transcriptome and proteome information for T4 phage and *E. coli* genes of interest. Thereby, we hope to provide a valuable resource to build and support hypotheses and biochemical studies on T4 phage infection regulation and related subjects. 

In addition, we set out to compare our transcriptome- and proteome-based classifications of T4 phage genes to obtain an overall impression of the correlation between the T4 phage transcriptome and proteome. To our surprise, we detected a large discrepancy between the fractions of T4 genes assigned to the individual infection phases. Most genes were classified as early transcripts based on our transcriptomics data, whereas our proteome-based classification yielded a comparably even distribution among T4 phage gene classes (Figure 4e–g). Thus, early T4 mRNAs encode early, middle, as well as late T4 proteins (Figure 4e). This is also emphasized in T4 genomic maps showing that clusters of early transcripts contribute to all classes of T4 phage proteins (Supplementary Figure S14). Late proteins are derived from all classes of T4 mRNAs (Figure 4e–g), while all late mRNAs only encode for a subset of late T4 proteins (Figure 4g). This discrepancy may be based on the expression principles of early T4 mRNAs in our transcriptomic data. For all early RNAs, moderate expression is detected already early during infection. However, early RNAs diverge into two distinct sets—one set with an expression peak in the early or middle infection phase and another set with a constitutively increasing expression to the end of the infection (Figure 2c). The finding of early mRNAs giving rise to late proteins (summarized in Supplementary Table S4) is in good agreement with the scientific concept of the post-transcriptional regulation of T4 phage gene expression, which has previously been built on reductionist biochemical studies [16,19]. Here, we were able to characterize this phenomenon globally. Still, it remains to be investigated how the entirety of early T4 mRNAs are regulated on a post-transcriptional and translational level to be translated late during infection. So far, it is well established that RegB specifically degrades early transcripts to shut down their translation [17,78]. This could also apply to the transcripts of late genes, which are transcribed early during infection.

In order to further elucidate reasons for discrepancies in the temporal appearance of phage transcripts and respective proteins, translational regulatory factors, such as the position and composition of the Shine–Dalgarno sequence, the involvement of ribosome-binding proteins, or the presence of riboswitches, still need to be studied in detail in future studies.

## 4. Conclusions

In this work, we described the first time-resolved dual-transcriptome and -proteome study of the T4 phage infection of *E. coli*, allowing for comprehensive molecular insights into the infection process. In this study, we confirmed that *E. coli* transcriptome is largely degraded during the infection, including both mRNAs and host tRNAs. Intriguingly, we identified four *E. coli* non-coding RNAs, which appear to possess unexpected stability throughout the infection. In stark contrast to the dual-transcriptome, the proteome of the host remains stable throughout infection, probably due to the utilization of the *E. coli* proteins by the T4 phage. Moreover, we could gain insights into the transcriptome and—for the first time—the comprehensive proteome of T4 phage infection, demonstrating the temporal control of T4 phage genes on two levels of gene expression. Correlating the phage transcriptome and proteome showed that specific T4 phage mRNAs and proteins are temporally decoupled, suggesting post-transcriptional and translational regulation mechanisms. Thus, we obtained the first global picture of T4 phage infection on the levels of gene expression, focusing on the phage and the host. Our data are in good agreement with multiple studies dedicated to the gene expression regulation of T4 phage infection. 

Moreover, we speculated that the temporal appearance of T4 phage proteins might be linked to their function. Surprisingly, one-third of T4 phage proteins are annotated as uncharacterized/hypothetical proteins. Based on our data sets, these hypothetical proteins might be functionally classified.

Thereby, this work is a valuable resource for future studies focusing on yet unexplored phage–host interactions and gene regulatory events during T4 phage infection. Moreover, this work exemplifies the power of time-series transcriptomics and proteomics to obtain a comprehensive understanding of gene expression during phage infection. Our data sets can explain observed changes in protein and transcript abundances and provide insights into the causal flow of molecular information during phage infection.

## Data Availability

Raw RNA-Seq data is deposited in GEO and accessible via identifier GSE211026. The MS Raw data can be accessed via the PRIDE/ProteomeXchange consortium under the project identifier PXD035873. POTATO4 is a user-friendly web application and accessible via https://rshiny.gwdg.de/apps/potato4/.

## Supplementary Materials

The following supporting information can be downloaded at: https://www.mdpi.com/article/10.3390/v14112502/s1, Figure S1: Fractions of *E. coli* tRNAs and rRNAs among all reads in all samples; Figure S2: Total RNA concentration per time point and replicate after RNA isolation; Figure S3: Principal component analysis of dual-transcriptome RNA-Seq samples; Figure S4: Global degradation of host transcripts during T4 phage infection; Figure S5: Time-resolved expression of transfer RNAs during T4 phage infection; Figure S6: Read coverage on genes of comparably stable *E. coli* transcripts during T4 phage infection; Figure S7: Northern blot analysis of *E. coli* and T4 phage transcripts during infection; Figure S8: Differential expression of *E. coli* genes during the early phase of infection; Figure S9: Initial analysis of the time-resolved dual-proteome of T4 phage infection of *E. coli*; Figure S10: Analysis of the correlation between all proteomics samples of this study; Figure S11: Normalized histograms of LFQ values contributed by phage and host; Figure S12: SDS-PAGE analysis of the *E. coli* and T4 phage proteome over the time course of infection; Figure S13: Relative abundance of T4 phage proteins from different functional groups; Figure S14: Genomic maps of T4 phage genome based on transcriptomic- and proteomic-based classifications of T4 phage genes; Table S1: DESeq2 analysis of RNA-Seq data; Table S2: Summary of the identified and classified T4 phage transcripts and proteins; Table S3: MaxQuant protein groups; Table S4: T4 phage genes grouped by transcriptome- and proteome-based classification and their corresponding GO terms.
