# Supplementary material for: Integrated Omics Reveal Time-Resolved Insights into T4 Phage Infection of E. coli on Proteome and Transcriptome Levels
Source: Viruses. 2022 Nov 12;14(11):2502. doi: 10.3390/v14112502 (PMC9697503; doi:10.3390/v14112502)
Supplement: Supplementary file 1 [file viruses-14-02502-s001.zip › Supplementary_Information_revised.pdf]

Supplementary Information

Integrated Omics Reveal Time-Resolved Insights into T4 Phage Infection of E. coli on Proteome and Transcriptome Levels

Maik Wolfram-Schauerte[1,†], Nadiia Pozhydaieva[1,†], Madita Viering[1], Timo Glatter[1], Katharina Höfer[1,*]

[1] Max-Planck-Institute for Terrestrial Microbiology, 35043 Marburg, Germany
[*] Correspondence: katharina.hoefer@synmikro.mpi-marburg.mpg.de; Tel.: +49-6421-28-216-24
[†] These authors contributed equally to this work.

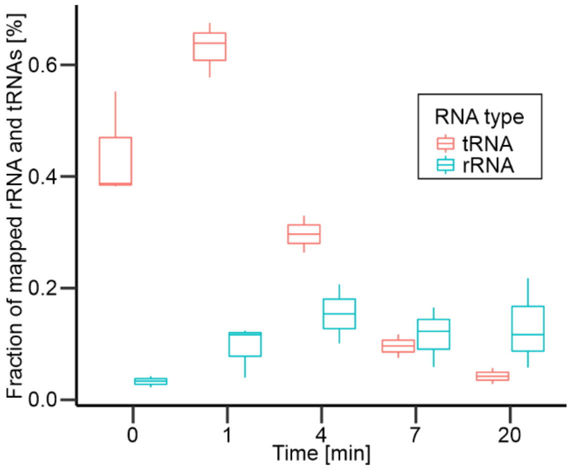

Supplementary Figure S1: Fractions of *E. coli* tRNAs and rRNAs among all reads in all samples.

Fractions of *E. coli* rRNAs (22, blue) and *E. coli* tRNAs (84, red) among all reads per sample were calculated from TPM normalized count data and visualized in a boxplot. Fractions of respective RNA groups are shown for each time point (0 min (before infection) and 1, 4, 7, 20 min post infection). Data at each time point is based on TPM values from biological triplicates (n = 3).

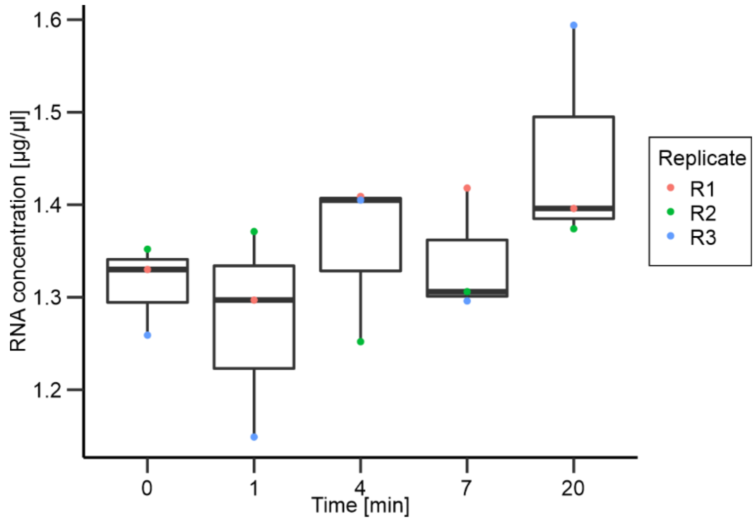

Supplementary Figure S2: Total RNA concentration per time point and replicate after RNA isolation. Total RNA was isolated, DNase I digested and resuspended in 200 µl RNase-free water. RNA concentrations were determined with the NanoDrop. Here, the concentrations are presented in a boxplot in order to visualize overall RNA yields for all samples [µg/µl]. RNA samples reflect the total RNA of T4 phage infection at 0 (before infection) and 1, 4, 7 and 20 min post-infection in biological triplicates (n = 3).

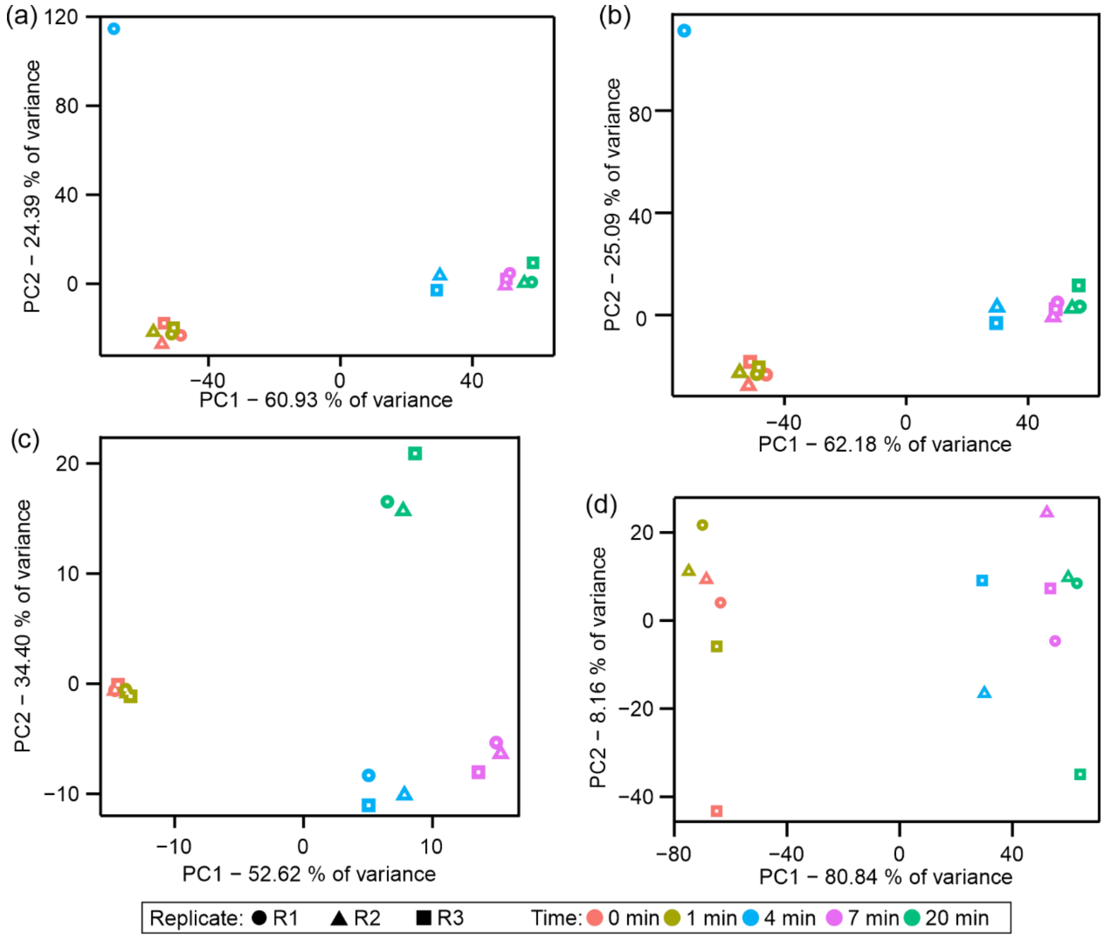

Supplementary Figure S3: Principal component analysis of dual-transcriptome RNA-Seq samples. Principal component analysis (PCA) of RNA-Seq samples based on read counts for *E. coli* and T4 phage genes. PCA analysis is shown for TPM normalized read counts for *E. coli* and T4 phage genes (**a**), *E. coli* genes only (**b**) and T4 phage genes only (**c**). Replicate 1 from 4 min time point (t4 R1) showed large discrepancies in *E. coli* gene expression compared to all other samples (**a, b**), whereas expression of T4 phage genes was in-line with the other replicates 2 and 3 from t4 (**c**). Thus, t4 R1 was excluded from further analyses which resulted in close clustering of replicates of the same time points in PCA of TPM normalized read counts for *E. coli* and T4 phage genes (**d**). Each PCA was performed on the basis of TPM normalized reads. Color indicates time point of infection, shape determines replicate. Principal components 1 (PC1) and 2 (PC2) with respective explained fraction of data variance are presented on x- and y-axis.

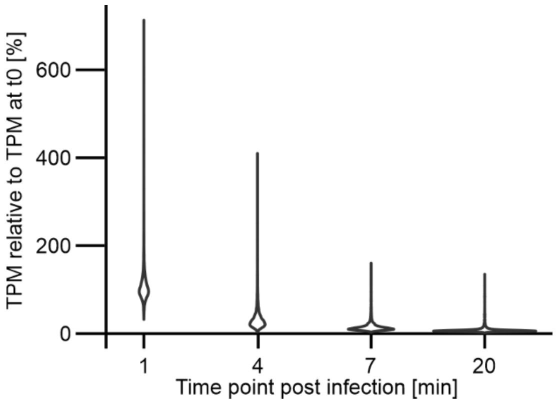

Supplementary Figure S4: Global degradation of host transcripts during T4 phage infection.

E. coli transcript degradation monitored with violin plots. Per time point (1, 4, 7 and 20 minutes post infection) the TPM for each *E. coli* gene and replicate were normalized by their mean TPM values at t0 (0 min post infection, uninfected *E. coli*). The violin plots display the distribution of these fractions per gene and time point post infection showing a decline of TPM values relative to t0. Calculations are based on mean TPM values from biological triplicates (n = 3) except for 4 min post-infection, where only duplicates (n = 2) were considered.

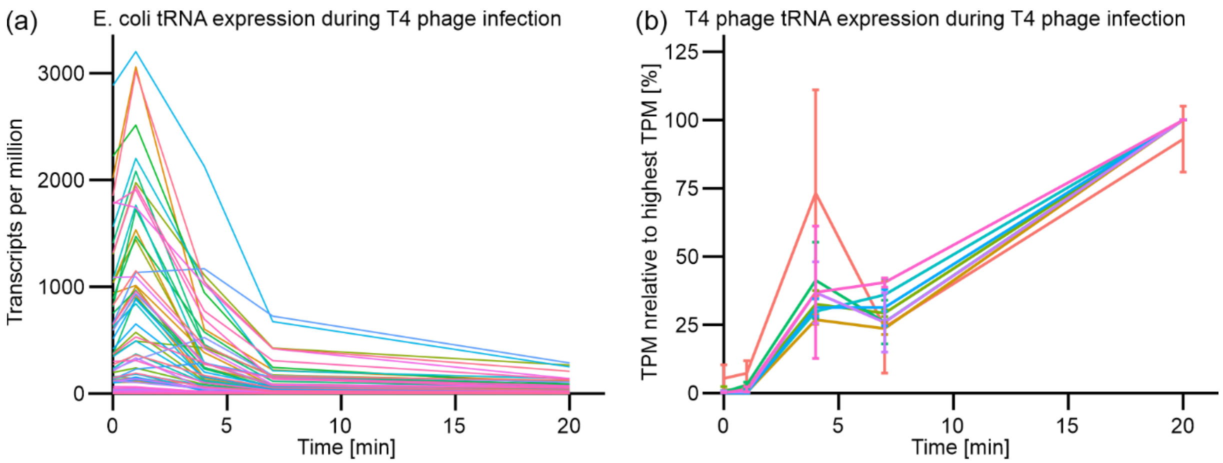

Supplementary Figure S5: Time-resolved expression of transfer RNAs during T4 phage infection.

a) Expression of transfer RNAs (tRNAs) derived from the host *E. coli* during T4 phage infection plotted as nominal mean TPM values which were calculated on the basis of TPM values from biological triplicates (n = 3; except for 4 min post infection, n = 2). **b**) Expression of T4 phage tRNAs over the time course of infection measured as the fraction of TPM values relative to the highest TPM value per replicate per T4 phage tRNA. Data points represent mean, normalized TPM values, errorbars are based on the standard deviation of data in biological triplicates (n = 3; except for 4 min post infection, n = 2). Each color represents a single tRNA from either *E. coli* or T4 phage.

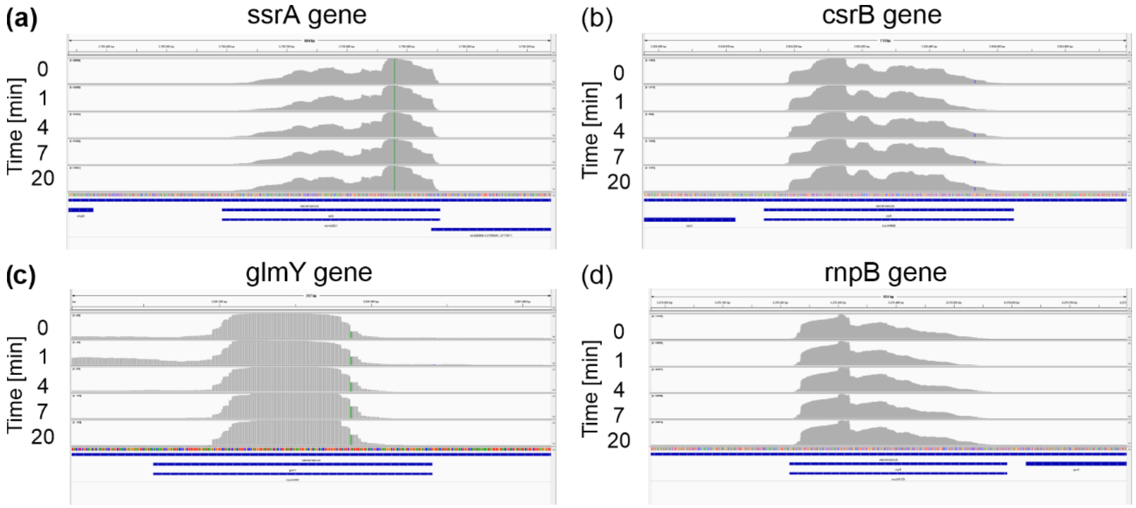

Supplementary Figure S6: Read coverage on genes of comparably stable *E. coli* transcripts during T4 phage infection. Read coverage on genes encoding for comparably *E. coli* stable transcripts under T4 phage infection. Coverages are depicted for all time points (0, 1, 4, 7 and 20 minutes) based on data for replicate 2 (R2) for the genes *ssrA* (a), *csrB* (b), *glmY* (c) and *rnpB* (d). Coverage visualization was performed using the Integrative Genomics Viewer at default settings.

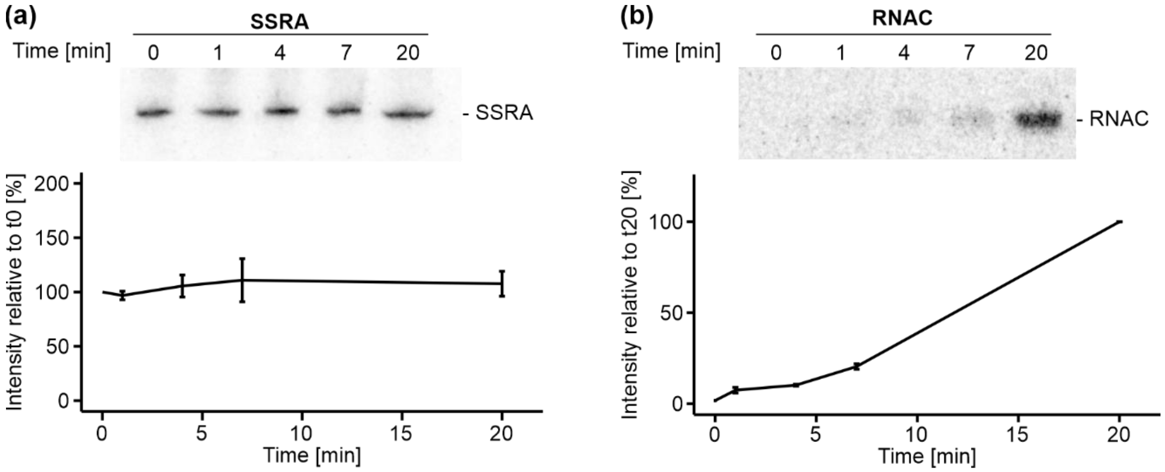

Supplementary Figure S7: Northern blot analysis of *E. coli* and T4 phage transcripts during infection. Northern blot analysis of SSRA transcripts over the time course of T4 phage infection at 0 min (before infection) and 1, 4, 7 and 20 minutes post infection shown for the *E. coli* SSRA transcript (**a**, upper panel) and the T4 phage sRNA RNAC (**b**, upper panel). Northern blot analysis was performed with 10 µg total RNA per time point. Band intensities were quantified using ImageLab 6.1 and normalized to either t0 (SSRA, **a**, lower panel) or t20 (RNAC, **b**, lower panel). Plots of normalized band intensities are based on three (n = 3, SSRA) or two (n = 2, RNAC) independent biological replicates of the Northern blots.

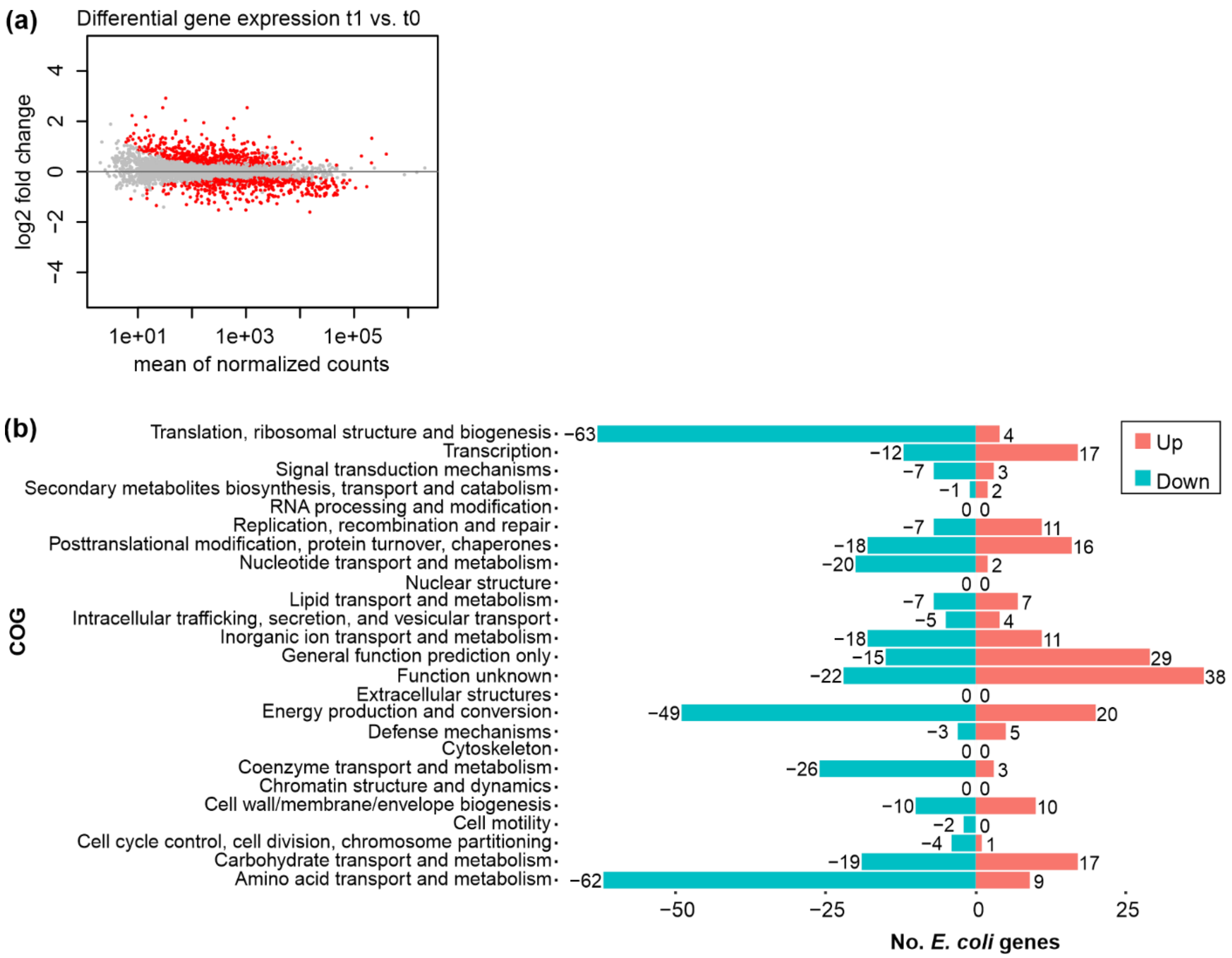

Supplementary Figure S8: Differential expression of *E. coli* genes during the early phase of infection. **a**) MA plot showing log2 fold changes of *E. coli* genes at 1 min post-infection compared to 0 min time point. Genes differentially expressed (LFC > 0 or > 0 and adjusted p-value < 0.05) are colored red. Differential expression analysis was performed with DESeq2. Therefore, T4 phage genes were removed from raw read count data, a pseudocount (0.5) was added and low expression and rRNA genes were removed for DESeq2 analysis. **b**) Assignment of significantly, differentially expressed *E. coli* genes to Clusters of Orthologous Groups (COGs) differentiating downregulated (cyan) or upregulated (red) *E. coli* genes during the early phase of T4 phage infection (1 min post-infection). The bar height determines the number of differentially expressed assigned to the respective COG.

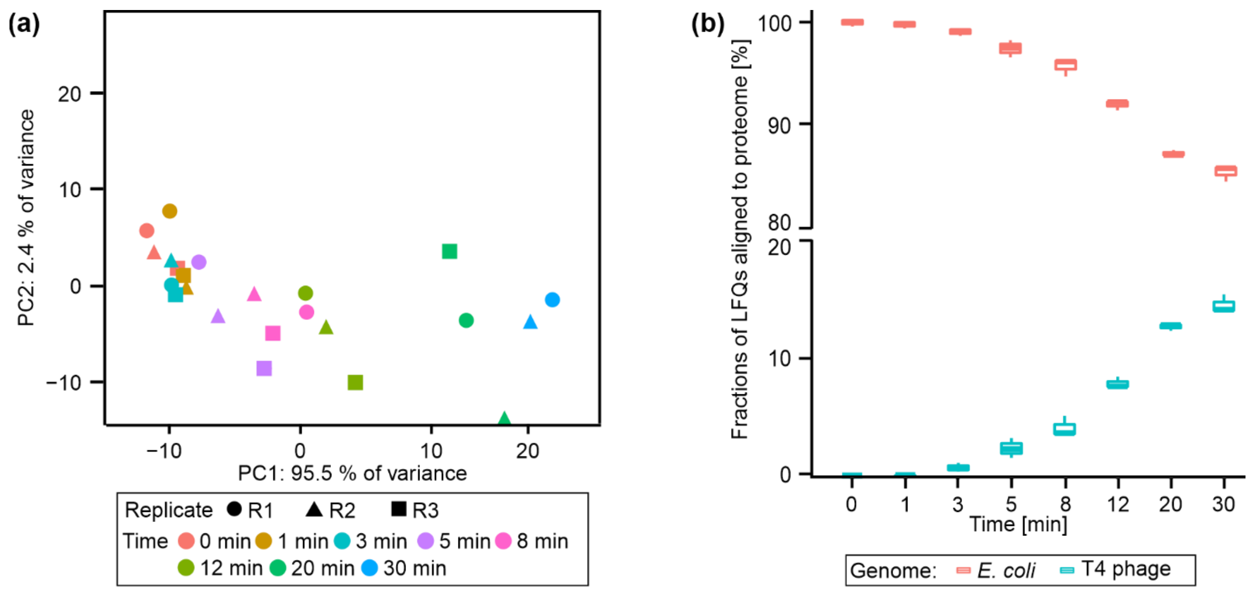

Supplementary Figure S9: *Initial analysis of the time-resolved dual-proteome of T4 phage infection of* E. coli. **a**) Principle component analysis (PCA) of proteomics samples based on LFQ values for individual *E. coli* and T4 phage proteins. The color indicates the time point of infection, the shape determines the replicate. Principal components 1 (PC1) and 2 (PC2) with a respective explained fraction of data variance are presented on the x- and y-axis. A close clustering of biological replicates per time point is observed (n = 3). **b**) Fractions of LFQ values derived from either the *E. coli* or the T4 phage proteome relative to the sum of all LFQ values per sample over the time course of T4 phage infection, based on biological triplicates (n=3).

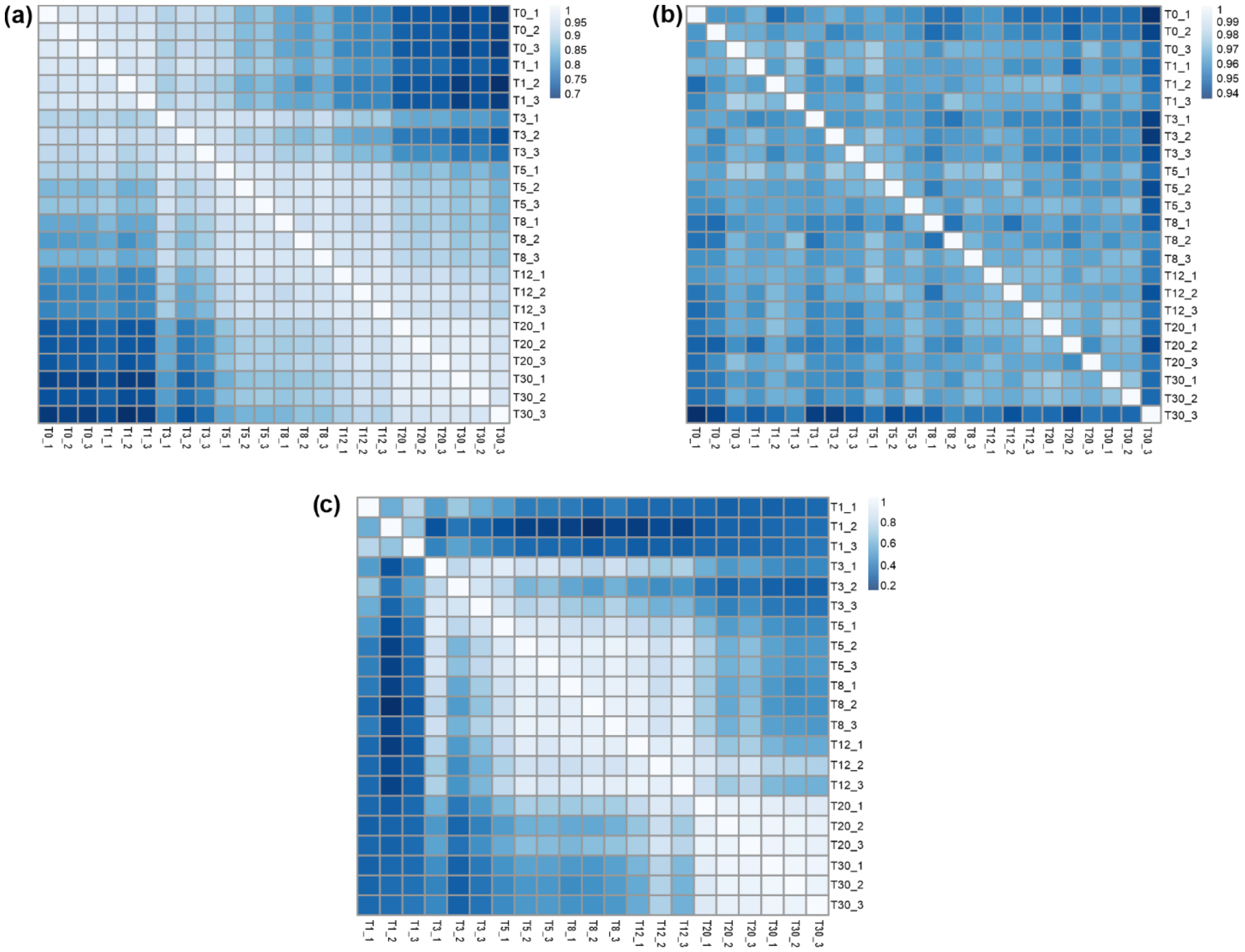

Supplementary Figure S10: Analysis of the correlation between all proteomics samples of this study. **a**) Pearson correlation between not processed samples (Pearson correlation coefficient: 0.7-1) presented in a heatmap. **b**) Pearson correlation between the analyzed samples in regard of *E. coli* proteins only presented in a heatmap. Low variation in Pearson correlation coefficient (0.94-1) confirms the stability of the *E. coli* proteome. **c**) Pearson correlation between the analyzed samples in regard of T4 phage proteins only presented in a heatmap. High variation in the Pearson correlation coefficient (0.2 -1) is in accordance with the dynamic expression of the T4 phage proteins. Samples are abbreviated by combining the respective time point (e.g. T12 = 12 min post-infection) and the biological replicate (e.g. R2 = replicate 2) to, e.g. T12_R2.

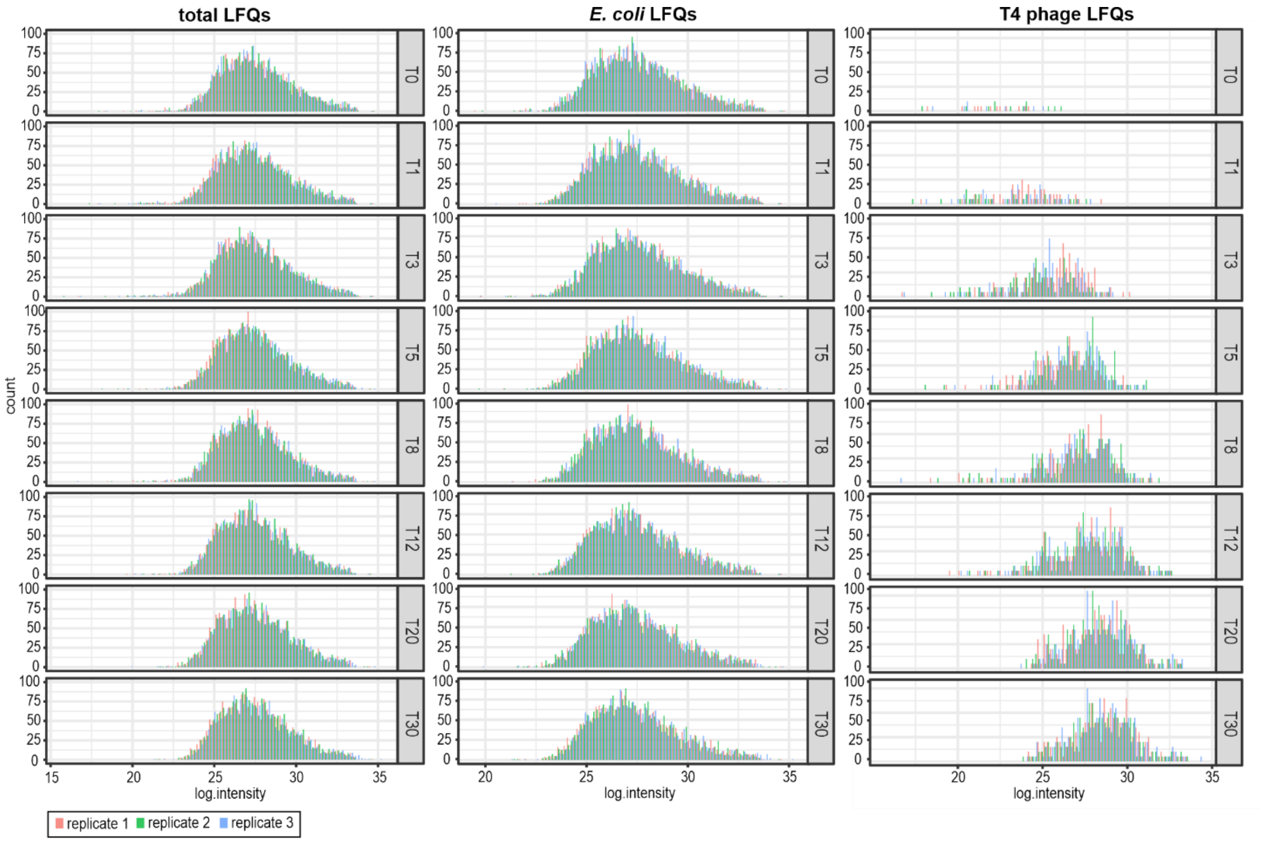

Supplementary Figure S11: *Normalized histograms of LFQ values contributed by phage and host.* Histograms of LFQ values are shown for all time points (T0, T1, T3, T5, T8, T12, T20, T30; e.g. T12 = 12 min post-infection) including all biological replicates (n = 3) per time point. Histograms are shown for all *E. coli* and host proteins (left panel), *E. coli* proteins only (middle panel) and T4 phage proteins only (right panel). All the histograms show normal distribution of the data, except for the histograms for T4 phage LFQs at early time points of infection (T0 – T3). This can be explained by the low abundance of phage proteins at the early stages of the infection.

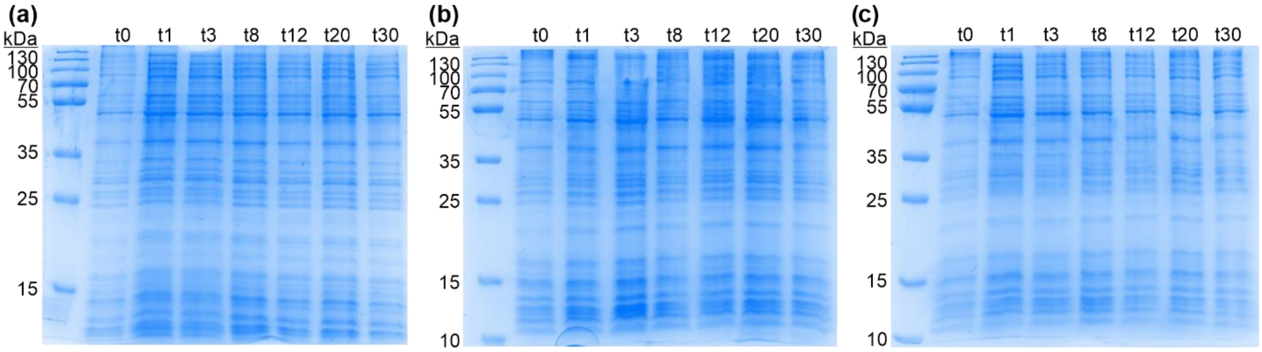

Supplementary Figure S12: *SDS-PAGE analysis of the* E. coli *and T4 phage proteome over the time course of infection.* Protein samples were taken prior to (t0) and 1 (t1), 3 (t3), 8 (t8), 12 (t12), 20 (t20) and 30 (t30) minutes after T4 phage infection of *E. coli* and subjected to SDS-PAGE analysis followed by protein visualization by Coomassie staining. No significant changes over the time course of infection can be observed. The analysis was performed in biological triplicates (**a**, **b**, **c**).

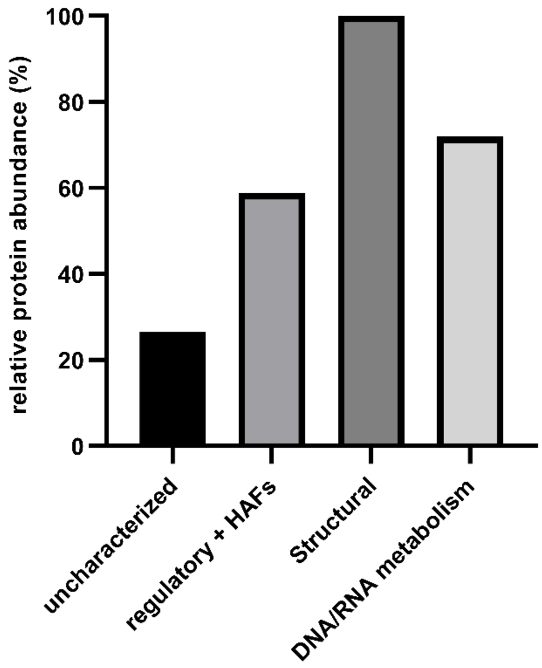

Supplementary Figure S13: *Relative abundance of T4 phage proteins from different functional groups.* Bar plot illustrating the abundance of four major T4 phage protein groups (displayed on x-axis) relative to the most abundant group (structural T4 phage proteins). Absolute abundance of protein groups was calculated as the sum of mean LFQ values for all proteins belonging to a specific functional group. Absolute abundances were normalized to the absolute abundance of structural proteins. Presented data is based on LFQ values from biological triplicates (n = 3).

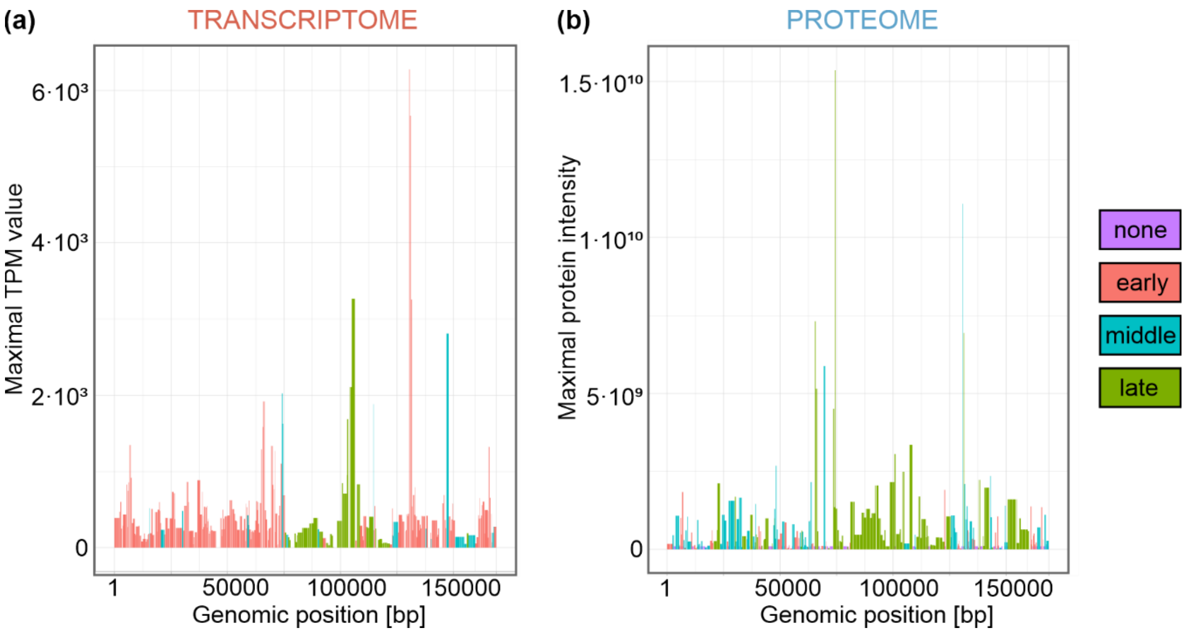

Supplementary Figure S14: Genomic maps of T4 phage genome based on transcriptomic- and proteomic-based classifications of T4 phage genes. **a**) Transcriptome-based classification showing bar height based on maximal TPM value for a T4 phage gene. **b**) Proteome-based classification showing bar height based on maximal LFQ value for a T4 phage gene-derived protein. X-axis represents the T4 phage genome coordinate in base pairs (bp). Each T4 phage gene is represented by a bar colored according to the infection phase it was classified to. Thus, early T4 transcripts contribute to all classes of T4 phage proteins and make up a major fraction of T4 phage genes. In contrast to that, the proteome is dominated by middle and late T4 phage proteins.
